# Long-Range Drone Detection of 24 G FMCW Radar with E-plane Sectoral Horn Array

**DOI:** 10.3390/s18124171

**Published:** 2018-11-28

**Authors:** Byunggil Choi, Daegun Oh, Sunwoo Kim, Jong-Wha Chong, Ying-Chun Li

**Affiliations:** 1 Collaborative Robots Research Center, Daegu Gyeongbuk Institute of Science and Technology, Daegu 42988, Korea; choibk@dgist.ac.kr (B.C.); dgoh@dgist.ac.kr (D.O.); 2 Department of Electronic Engineering, Hanyang University, Seoul 04763, Korea; remero@hanyang.ac.kr (S.K.); jchong@hanyang.ac.kr (J.-W.C.)

**Keywords:** FMCW, drone detection, 3D subspace-based algorithm

## Abstract

In this work, a 24-GHz frequency-modulated continuous-wave (FMCW) radar system with two sectoral horn antennas and one transmitting lens antenna for long-range drone detection is presented. The present work demonstrates the detection of a quadcopter-type drone using the implemented radar system up to a distance of 1 km. Moreover, a 3D subspace-based algorithm is proposed for the joint range-azimuth-Doppler estimation of long-range drone detection. The effectiveness of the long-range drone detection is verified with the implemented radar system through a variety of experiments in outdoor environments. This is the first such demonstration for long-range drone detection with a 24-GHz FMCW radar.

## 1. Introduction

The use of civilian drones has stirred public concern in recent years due to the threat to public safety and national security, and civilian drone surveillance has become a very important but largely unexplored topic [1,2,3]. Many efforts have been made in terms of drone detections based on various techniques, such as audio detection/classification of drones in [4,5,6] and the use of cameras for the movements of drones in [7,8,9]. This work focused on a radar detection technique for long-range drone detection utilizing radar systems.

Generally, the small drones have a few pairs of rotor blades, and the blades rotate when a drone is flying. The micro-scale movements of blades produce additional Doppler shifts, referred to as micro-Doppler feature [10]. Due to the rotation of blades, the phenomenon of “blade flashes” will be captured by analyzing the micro-Doppler features [11]. The “blade flashes” is induced by the strong reflection of rotor blades when a pair of blades are momentarily aligned normal to the radar beam. Thus, the analysis of micro-Doppler features is always utilized for drone classification [12,13]. As presented in [14], the micro-Doppler signatures including blade flashes due to the propeller blades were observed from a drone “DJI Phantom 3 Standard” using a 94-GHz FMCW radar at a range of 120 m. Instead of FMCW radar, an X-band CW radar at 9.7-GHz in [15] was used to collect data for the micro-Doppler feature analysis of drones, and the distances between the radar and targets are limited from 3 m to 150 m. In [16], a coherent pulsed radar NetRAD operating at 2.4 GHz was utilized for data collection to extract the micro-Doppler features of drones at ~60 m from the baseline. The work [17] aims to classify the birds and drones by using the BirdRad radar system working at 3.25 GHz, and the observations of drones were done at a range of 300 m to 400 m. In [18], a distributed FMCW radar system with a transmitter and a receiver was proposed to detect drones within a 500 m range, and only the range and Doppler of drones were obtained through 2D FFT processing. The conventional radar systems in [14,15,16,17] organized the drone classification successfully, but the effective detection range of drones are limited to a few meters. Although the radar system in [17,18] can detect the drones in a longer range from 300 m to 500 m, only micro-Doppler or range and Doppler information are estimated by micro-Doppler feature analysis or simple FFT processing. However, the effective detection of drones in long range and the three-dimensional (3D) parameter estimation of range/azimuth/Doppler for the detected drones are important.

In this work, a 24-GHz FMCW radar system is implemented with a transmitting lens horn antenna and a two-element receiving sectoral antenna array, for the joint range-azimuth-Doppler 3D detection of drones over a long range up to 1 km. The FMCW signals should be transmitted with a high power and a high gain. Thus, the power amplifier (PA) circuit is designed to achieve 38 dBm output with 4 PA chips of 34 dBm output P1. The PA output signals of 38 dBm are emitted by the high gain lens horn antenna of 25 dBi. Moreover, the receiving antenna array, with the element spacing one lambda of 24 GHz signals, are also designed in a form of E-plane sectoral horn antennas [19] to achieve a high gain of 14 dBi. Base on the designed two-element sectoral horn antenna array, a 3D subspace-based algorithm is proposed for the joint range-azimuth-Doppler 3D estimation of the long-range drones. In Section 2, we give the system model for the proposed algorithm and implemented radar system. In Section 3, we present the conventional algorithms. In Section 4, we demonstrate the proposed algorithms. In Section 5, we demonstrate the implementation of the designed radar system. In Section 6, the conducted experiments and experiment results are presented. We summarize our results and discuss future work in Section 7.

Several experiments in outdoor environments were conducted, and the good performance of the modified 3D subspace-based algorithm and the implemented 24-GHz FMCW radar system with a transmitting lens antenna and two receiving sectoral horn antennas was verified experimentally.

## 2. System Model

In our implemented system, the transmitted *P* FMCW chirp pulses can be defined by:

(1) $$\begin{array}{l} s_{p}(t)=\sum_{p=0}^{P-1}s\left(t-pT_{PRI}\right)\\ \mathrm{where}\;s(t)=\{\begin{array}{ll} \exp\left[j\left(2\pi f_{c}t+\frac{\mu }{2}t^{2}\right)\right] & \;\mathrm{for}\;0\leq t<T_{sym}\\ \;0 & \;\mathrm{elsewhere}\; \end{array}, \end{array}$$

where *p* = 0, 1, …, *P* − 1, *T_PRI_* is the pulse repetition interval (PRI), *f_c_* denotes the carrier frequency, *μ* is the rate of change of the instantaneous frequency of a chirp signal, and *T_sym_* is the duration of the FMCW chirp pulse. Then the bandwidth *B* of the transmitted FMCW chirp pulses can be obtained by *B* = *μ*/*T_sym_*. For one observation of the targets, *P* pulses are collected for parameter estimation.

**Figure 1:**
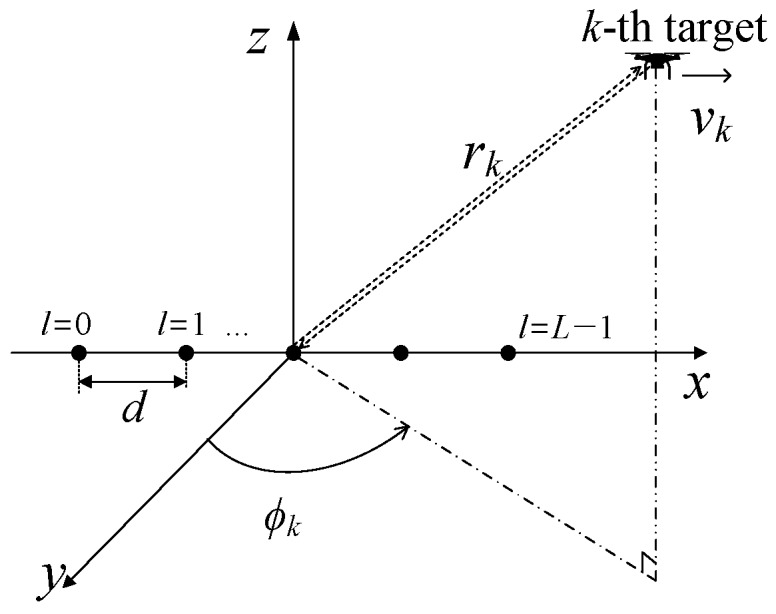
Radar system scenario with the two-element sectoral horn antenna array.

As seen in Figure 1, we assume that the reflected signals from the *K* targets arrive at the receiving sectoral horn array with (*ϕ_k_*, *v_k_*, *τ_k_*), *k* = 0, 1, …, *K* − 1, where *ϕ_k_*, *v_k_*, and *τ_k_* are the azimuth angle, radial velocity, and time delay of the *k*-th target, respectively. We define *x_l_*_,*p*_(*t*) as the received signal of the *p*-th pulse on the *l*-th antenna element, *l* = 0, 1, …, *L* − 1. Thus, the signal representation of the two-element sectoral antenna array can be modeled as:

(2) $$x_{l,p}(t)=\sum_{k=0}^{K-1}a_{k}\exp\left(-j\frac{2\pi ld\sin\phi_{k}}{\lambda }\right)s_{p}\left(t-\tau_{k}\right)+w_{l,p}(t),$$

where *a_k_* is the complex response of the antenna to the *k*-th receiving signal, *d* is the spacing between the sensors, *λ* denotes the wave length, *c* is the propagation speed of the wavefronts, and *w_l_*_,*p*_ is the additive white Gaussian noise (AWGN) of the *p*-th pulse at the *l*-th antenna.

The received FMCW signals can be transformed into a sinusoidal waveform by the de-chirp operation [20], which involves multiplication of the received signal with a transmitted chirp replica (the reference signal). The obtained sinusoidal waveforms are called beat signal as in [21], and it can be represented after the de-chirp operation by:

(3) $$\begin{array}{l} y_{l,p}\left(t\right)=x_{l,p}\left(t\right)s\left(t\right)\\ =\sum_{k=0}^{K-1}a_{k}\exp\left(j\frac{\pi }{\lambda }ld\sin\phi_{k}\right)\exp\left(j\left(f_{c}t+2\pi f_{c}\tau_{k}-\frac{\mu }{2}\tau_{k}^{2}\right)\right)+{\bar{w}}_{l,p}(t) \end{array}$$

where ${\bar{w}}_{l,p}(t)$ denotes the transformed AWGN signal. Assuming that the *k*-th target in the direct path distance *R_k_* away from the antenna is moving with a constant radial velocity *v_k_* over *P* pulses, the direct path distance between the antenna and the *k*-th object for the *p*-th pulse is changed to *R_p,k_* = *R_k_* + *v_k_pT_PRI_* = *R_k_* + *v_k_t*. Thus, the time delay *τ_k_* of the *k*-th target for the *p*-th pulse can be obtained by:

(4) $$\tau_{k}=\frac{2R_{p,k}}{c}=\frac{2\left(R_{k}+v_{k}t\right)}{c},$$

We substituting Equation (4) in Equation (3), and the Equation (3) is rewritten with some approximation of [22] by:

(5) $$y_{l,p}\left(t\right)=\sum_{k=0}^{K-1}\left(\begin{array}{l} a_{k}\exp\left(j\frac{\pi }{\lambda }ld\sin\phi_{k}\right)\\ \times \exp\left(j\left(2\pi f_{c}\frac{2\left(R_{k}+v_{k}t\right)}{c}+\frac{2\mu R_{k}}{c}t+\gamma_{k}\right)\right) \end{array}\right)$$

where $\gamma_{k}=\frac{2Bv_{k}}{T_{sym}c}t^{2}$ denotes the Range-Doppler-Coupling and can be neglected as described in [15].

After analog-to-digital conversion, the discrete time model for *y_l,p_* (*t*) of (3) with the sampling frequency *f_s_* = 1/*T_s_* satisfying the Nyquist criterion can be derived by *y_l,p_*[*n*] = *y_l,p_*(*nT_s_*) for *p* = 0, …, *P* − 1, *l* = 0, …, *L* − 1, and *n* = 0, …, *N* − 1, where *N* = *T_sym_*/*T_s_*. Thus, each of the signals received by the *l*-th antenna elements can be represented by

(6) $$Y_{l}=\left[\begin{array}{cccc} y_{l,0}\left[0\right] & y_{l,0}\left[1\right] & \cdots & y_{l,0}\left[N-1\right]\\ y_{l,1}\left[0\right] & y_{l,1}\left[1\right] & \cdots & y_{l,1}\left[N-1\right]\\ \vdots & \vdots & \vdots & \vdots \\ y_{l,P-1}\left[0\right] & y_{l,P-1}\left[1\right] & \cdots & y_{l,P-1}\left[N-1\right] \end{array}\right]$$

**Figure 2:**
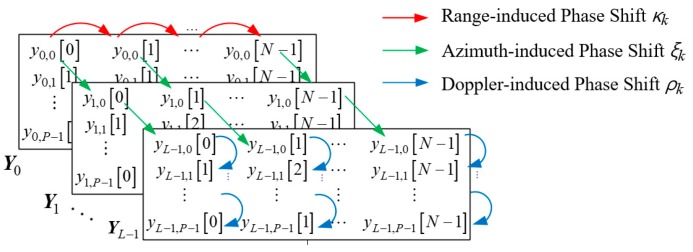
Illustration of the 3D phase shifts.

Then, there are three kinds of phase shifts in the received signal model, as depicted in Figure 2.

The three kinds of phase shifts, range-induce phase shift *κ_k_*, azimuth-induced phase shift *ξ_k_*, and Doppler-induced phase shift *ρ_k_*, can be defined from the obtained beat signal of Equation (3), as:

(7) $$\begin{array}{l} \kappa_{k}=\exp\left(j\frac{2\mu R_{k}}{c}T_{s}\right),\\ \xi_{k}=\exp\left(j\frac{\pi }{\lambda }d\sin\phi_{k}\right),\\ \mathrm{and}\;\rho_{k}=\exp\left(j\frac{4\pi f_{c}T_{PRI}}{c}v_{k}\right). \end{array}$$

## 3. Conventional Algorithms

In order to make it easy to understand the proposed 3D estimation algorithm in Section 4, the conventional 1D [23] and 2D [24] super-resolution algorithms by using correlation matrix are introduced in this section.

### 3.1. Conventional 1D Auto-Correlation Matrix

Conventional super-resolution techniques, such as MUltiple SIgnal Classification (MUSIC) [25] and ESPRIT [26], are based on eigen-decomposition of the auto-correlation matrix of the preceding signal model in Equation (3). For instance, in [23], the temporal auto-correlation matrix ***R****_T_* is utilized for range estimation, and it can be obtained from the sampled data of the *p*-th pluse received by the *l*-th antenna, here *p* = 1 and *l* = 1, as

(8) $$R_{T}=\sum_{n=0}^{N-L_{1}}y_{n}y_{n}^{H}$$

where

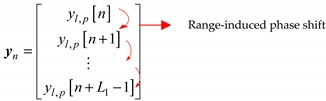
(9)

Here, *L*_1_ denotes the selection parameter satisfying 2 ≤ *L*_1_ < *N*. The sampled data of the *p*-th pluse received by the *l*-th antenna includes *N* elements as [*y_l_*_,*p*_[0], *y_l_*_,*p*_[1], …, *y_l_*_,*p*_[*N* − 1]], and they will be divided into *N* − *L*_1_ + 1 consecutive segments of length *L*_1_. The *n*-th segment include the elements [*y_l_*_,*p*_[n], *y_l_*_,*p*_[n + 1], …, *y_l_*_,*p*_[*n + L*_1_ − 1]] as shown in the Equation (9). To be specific, the first segment include the first *L*_1_ elements [*y_l_*_,*p*_[0], *y_l_*_,*p*_[1], …, *y_l_*_,*p*_[*L*_1_ − 1]], the second segment include the elements [*y_l_*_,*p*_[1], *y_l_*_,*p*_[2], …, *y_l_*_,*p*_[*L*_1_]], and so on, the last segment include the elements [*y_l_*_,*p*_[*N* − *L*_1_ − 1], *y_l_*_,*p*_[*N* − *L*_1_], …, *y_l_*_,*p*_[*N* − 1]]. The range-induced phase shift can be obtained by the multiplication of the adjacent elements of *y_n_*, for example, *y_l_*_,*p*_[0]y*_l__,*p*_[1]. [•]* denotes the conjugation of the data contained within. Therefore, the temporal auto-correlation matrix ***R****_T_* includes the range-induced phase shifts. Then, the matrix ***R****_T_* can be decomposed by eigenvalue decomposition (EVD), and the conventional 1D MUSIC algorithm is adopted for range estimations. The steering vector of the 1D MUSIC algorithm can be defined by:

(10) $$s_{q}=\left[\begin{array}{cccc} 1 & \exp\left(j\frac{2\pi }{Q}q\right) & \cdots & \exp\left(j\frac{2\pi }{Q}\left(L_{1}-1\right)q\right) \end{array}\right].$$

The pseudo-spectrum is obtained by the MUSIC algorithm. By the peak detection method, the *K* peaks can be detected, and the indexes {*q*_0_, *q*_1_,…, *q*_*K*−1_}, at which the *K* peaks are found, are used for delay estimation based on the relationship in *κ_k_* of (4), such that:

(11) $${\hat{R}}_{k}=\frac{c}{2\mu }\left(\frac{q_{k}}{QT_{s}}\right).$$

This method is also effective for 1D angle estimation or 1D Doppler estimation.

### 3.2. Conventional 2D Auto-Correlation Matrix

The authors of [24] show that the 1D auto-correlation matrix was extended to the spatial-temporal auto-correlation matrix for 2D estimation of joint range and azimuth angle. The spatial-temporal auto-correlation matrix is defined based on the sampled sequences of the *p*-th pulse by:

(12) $$R_{S-T}=\sum_{l=0}^{L-L_{2}}\sum_{n=0}^{N-L_{1}}y_{l,n}y_{l,n}^{H},$$

where:

(13) $$y_{l,n}=\left[\begin{array}{c} m_{l,n}\\ m_{l+1,n}\\ \vdots \\ m_{l+L_{2}-1,n} \end{array}\right]\;\mathrm{where}\;m_{l,n}=\left[\begin{array}{c} y_{l,p}\left[n\right]\\ y_{l,p}\left[n+1\right]\\ \vdots \\ y_{l,p}\left[n+L_{1}-1\right] \end{array}\right].$$

In Equation (13), the vector ***y**_l_*_,_*_n_* includes two kinds of phase shift: range-induced phase shift between the elements *y_l_*_,*p*_[*n*] and *y_l_*_,*p*_[*n* + 1], and azimuth-induced phase shift between the elements *y_l_*_,*p*_[*n*] and *y_l_*_+1,*p*_[*n*]. Similar with the previous method in Section 3.1, the conjugation between the corresponding adjacent elements can be utilized for phase shift calculation. For example, the range-induced phase shift can be obtained by the multiplication *y_l_*_,*p*_[*n*]y^*^_l__,*p*_[*n* + 1], and the azimuth-induced phase shift can be obtained by the multiplication *y_l_*_,*p*_[*n*]y^*^_l__+1__,*p*_[*n*]. As shown in Equation (13), the vector ***y**_l_*_,_*_n_* consist of the stacked vectors ***m****_l_***_,_***_n_*, which make is possible to estimate the range-induce phase shift and azimuth-induced phase shift simultaneously. Further, there is a dual-shift-invariant structure in the proposed spatial temporal auto-correlation matrix ***R****_S_*_−*T*_, and we call the matrix ***R****_S_*_−*T*_ conceptually as a 2D matrix based on the parameter space (herein, range and azimuth). Similarly, the spatial temporal auto-correlation matrix in (12) can be factorized by EVD, and the corresponding 2D steering vector is defined such that

(14) $$\begin{array}{l} s_{2D}=s_{q}⊗s_{w}\\ =\left[\begin{array}{cccc} 1 & \exp\left(j\frac{2\pi }{Q}q\right) & \cdots & \exp\left(j\frac{2\pi }{Q}\left(L_{1}-1\right)q\right) \end{array}\right],\\ \;⊗\left[\begin{array}{cccc} 1 & \exp\left(j\frac{2\pi }{W}w\right) & \cdots & \exp\left(j\frac{2\pi }{W}\left(L_{2}-1\right)w\right) \end{array}\right], \end{array}$$

for *q* = 0, …, *Q* − 1 and *w* = −*W*/2, …, *W*/2−1, and ⨂ denotes the Kronecker product.

After the *K* peaks are detected from the obtained 2D pseudo-spectrum, the paired indexes ${\left\{q_{k},w_{k}\right\}}_{K-1}^{k=0}$ are obtained. Finally, the paired range and azimuth angle estimations can be estimated as

(15) $$\begin{array}{l} {\hat{R}}_{k}=\frac{c}{2\mu }\left(\frac{q_{k}}{QT_{s}}\right),\\ {\hat{\theta }}_{k}=\arcsin\left(\frac{\lambda w_{k}}{\pi dW}\right) \end{array}$$

Similarly, the conventional 2D auto-correlation matrix can also be utilized for range-Doppler estimation, azimuth-Doppler estimation, or azimuth-elevation angle estimation.

## 4. Proposed Algorithm

Since the proposed method was developed for joint estimation of range, azimuth angle and velocity for FMCW radar, we propose a 3D spatial-temporal auto-correlation matrix to reduce the processed matrix size.

**Figure 3:**
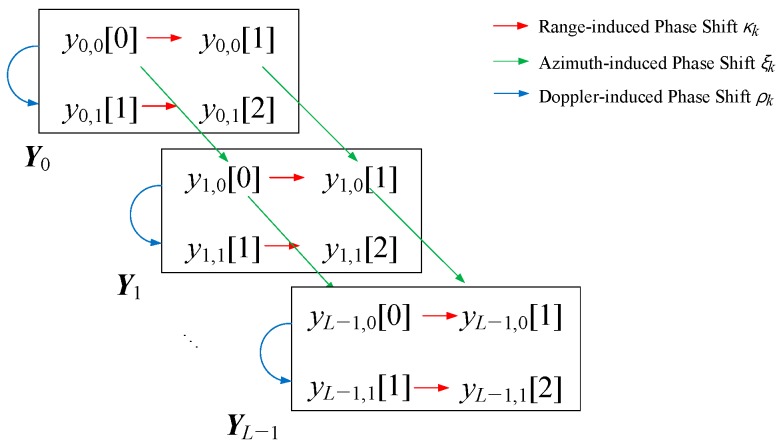
Simplified version of Figure 2.

As shown in Figure 2, there are three kinds of phase shifts in the received signal model. We put the simplified version Figure 3 here and make a further explanation on the 3D phase shifts. Each kind of phase shift can be calculated by the multiplication between the adjacent elements. For instance, the range-induced phase shift can be obtained by the multiplication of *y*_0,0_[0]y^*^_0,0_[1] and *y*_1,0_[0]y^*^_1,0_[1], the azimuth-induced phase shift can be obtained by the multiplication of *y*_0,0_[0]y^*^_1,0_[0] and *y*_1,0_[0]y^*^_L__−1,0_[0], and the Doppler-induced phase shift can be obtained by the multiplication of of *y*_0,0_[0]y^*^_0,1_[1] and *y*_1,0_[0]y^*^_1,1_[1]. The proposed method is developed based on 3D phase shifts by organizing the 3D steering vector and calculating the 3D pseudospectrum as shown in this section.

### 4.1. Proposed 3D Subspace-Based Algorithm

To jointly estimate the 3D parameters of range, Doppler, and azimuth angle, the proposed 3D auto-correlation matrix ***R*** is defined as:

(16) $$R=\sum_{l=0}^{L-L_{3}}\sum_{p=0}^{P-L_{2}}\sum_{n=0}^{N-L_{1}}y_{l,p,n}y_{l,p,n}^{H},$$

where

(17) $$y_{l,p,n}=\left[\begin{array}{c} M_{l,p,n}\\ M_{l+1,p,n}\\ \vdots \\ M_{l+L_{3}-1,p,n} \end{array}\right]\;\mathrm{where}\;M_{l,p,n}=\left[\begin{array}{c} m_{l,p,n}\\ m_{l,p+1,n}\\ \vdots \\ m_{l,p+L_{2}-1,n} \end{array}\right],m_{l,p,n}=\left[\begin{array}{c} y_{l,p}\left[n\right]\\ y_{l,p}\left[n+1\right]\\ \vdots \\ y_{l,p}\left[n+L_{1}-1\right] \end{array}\right]$$

Here, *L*_1_, *L*_2_, and *L*_3_ denote the selection parameters satisfying 2 ≤ *L*_1_ *< N*, 2 ≤ *L*_2_ *< P*, and 2 ≤ *L*_3_ < *N*, respectively. In Equation (17), the vector ***y**_l_*_,*p*,_*_n_* includes three kinds of phase shift: range-induced phase shift between the adjacent elements *y_l_*_,*p*_[*n*] and *y_l_*_,*p*_[*n* + 1], azimuth-induced phase shift between the adjacent elements *y_l_*_,*p*_[*n*] and *y_l_*_+1,*p*_[*n*], and Doppler-induced phase shift between the adjacent elements *y_l_*_,*p*_[*n*] and *y_l_*_,*p*+1_[*n*]. Similar with the previous method in Section 3.2, the range-induced phase shift can be obtained by the multiplication *y_l_*_,*p*_[*n*]y^*^_l__,*p*_[*n* + 1], the azimuth-induced phase shift can be obtained by the multiplication *y_l_*_,*p*_[*n*]y^*^_l__+1__,*p*_[*n*], and the Doppler-induced phase shift can be obtained by the multiplication *y_l_*_,*p*_[*n*]y^*^_l__,*p*+1_[*n*]. Therefore, the stacked spatial temporal auto-correlation matrix ***R*** consists of three kinds of phase shift: range-induced phase shift, azimuth-induced phase shift, and Doppler-induced phase shift. The matrix is ***R*** called 3D matrix conceptually due the three estimated parameters. Then, a 3D shift-invariant structure exits in the proposed 3D auto-correlation matrix ***R***, and the 3D auto-correlation matrix can be factorized using EVD by

(18) $$\begin{array}{l} R=U\Sigma U^{H},\\ \mathrm{where}\;U=\left[\begin{array}{cc} U_{s} & U_{n} \end{array}\right],\Sigma =\left[\begin{array}{cc} \Sigma_{s} & \Sigma_{n} \end{array}\right] \end{array},$$

where ***U****_s_* denotes the signal subspace of ***R***; ***U****_n_* denotes the noise subspace of ***R***; ***Σ****_s_* = [*σ*_0_, *σ*_1_, …, *σ_K_*_−1_] denotes the diagonal matrix having *K* eigenvalues, corresponding to the *K* column vectors of ***U****_s_*; and ***Σ****_n_* denotes the diagonal matrix having *L*_1_ × *L*_2_ × *L*_3_ − 1 eigenvalues, equivalent to the noise variance. After EVD on ***R***, the matrices ***U*** and ***Σ*** are given as pairs, [***U****_s_* ***U****_n_*] and [***Σ****_s_* ***Σ****_n_*], respectively. However, EVD cannot separate the signal subspace and noise subspace automatically. In this paper, the criterion of the minimum description length (MDL) [27] for separation of signal subspace and noise subspace is used, and the derived singular values in Equation (18) are processed by MDL to obtain the estimated number of targets $\hat{K}$.

The 3D steering vector for calculating the 3D pseudo-spectrum can be defined by:

(19) $$\begin{array}{l} s_{q}={\left[\begin{array}{cccc} 1 & \exp\left(j\frac{2\pi }{Q}q\right) & \cdots & \exp\left(j\frac{2\pi }{Q}\left(L_{1}-1\right)q\right) \end{array}\right]}_{1\times L_{1}},\\ s_{w}={\left[\begin{array}{cccc} 1 & \exp\left(j\frac{2\pi }{W}w\right) & \cdots & \exp\left(j\frac{2\pi }{W}\left(L_{2}-1\right)w\right) \end{array}\right]}_{1\times L_{2}},\\ s_{g}={\left[\begin{array}{cccc} 1 & \exp\left(j\frac{2\pi }{G}g\right) & \cdots & \exp\left(j\frac{2\pi }{G}\left(L_{3}-1\right)g\right) \end{array}\right]}_{1\times L_{3}} \end{array}$$

for *q* = 0, …,*Q* − 1, *w* = *−W*/2, …,*W*/2 − 1, and *g* = 0, …, *G* − 1, respectively.

(20) $${\mathrm{PseudoSpectrum}}_{3D}=\frac{1}{s_{q,w,g}^{H}U_{s}U_{s}^{H}s_{q,w,g}}$$

where ***s****_q_*_,*w*,*g*_ = *c****s****_q_* ⨂ ***s****_w_* ⨂ ***s****_g_*.

By the peak detection method, the *K* peaks can be detected for each 1D pseudo-spectrum searching, and the estimated three indexes ${\left\{q_{k}\right\}}_{k=0}^{K-1},{\left\{w_{k}\right\}}_{k=0}^{K-1},{\left\{g_{k}\right\}}_{k=0}^{K-1}$ at which the *K* peaks are found, such that

(21) $$\left\{q_{k},w_{k},g_{k}\right\}={\left\{\max_{k}\left[{\mathrm{PseudoSpectrum}}_{3D}\right]\right\}}_{k=0}^{K-1},$$

where max*_k_*[•] denotes the *k*-*th* biggest value. For example, the indexes {*q_k_*,*w_k_*,*g_k_*} is for the *k*-th peak of the 3D pseudo-spectrum.

Since three indexes of 3D pseudo-spectrum are estimated, estimations for ranges, azimuth angles and velocities of *K* targets can be organized by the relationship in *κ_k_*, *ξ_k_* and *ρ_k_* of in Equation (7), for *k* = 0, …, *K* − 1, respectively:

(22) $$\begin{array}{l} {\hat{R}}_{k}=\frac{c}{2\mu }\left(\frac{q_{k}}{QT_{s}}\right),\\ {\hat{\theta }}_{k}=\arcsin\left(\frac{\lambda w_{k}}{\pi dW}\right)\\ \mathrm{and}\;{\hat{v}}_{k}=\frac{cg_{k}}{4\pi f_{c}T_{PRI}G}. \end{array}$$

### 4.2. Comparision with the Previous Works

This paper proposed joint 3D estimation of range, azimuth angle and velocity of drones for FMCW radar. There already exist many previous works on the drone detection or classification through the radar system. The designed algorithm and radar system in this paper aims to detect the drones in long range up to 1 km, and the comparison between the previous works and this work is shown in Table 1. It is apparently that the implemented radar system can detect the drones in a longer range than the previous works.

## 5. Implementation of 24-GHz FMCW Drone Detection Radar

In this section, the structure of the implemented 24 GHz FMCW long range drone detection radar system will be introduced, including the transmitter (Tx) and receiver (Rx) antennas, 24-GHz transceiver and IF, data logging system, and data processing system. The data-logging platform logs and transmits the raw radar data to the PC, and then the saved raw radar data is processed by the implemented proposed algorithm with MATLAB 2016b of The MathWorks, Inc. (Natick, MA, USA).

### 5.1. Tx Lens Antenna and Rx Sectoral Horn Antennas

**Figure 4:**
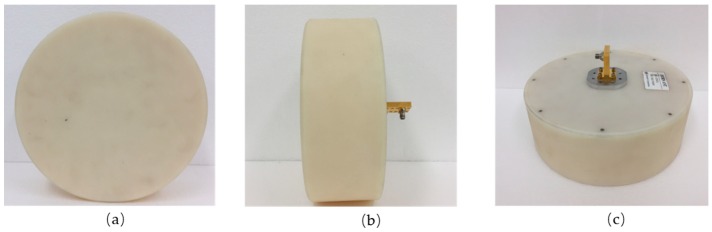
Illustration of Tx lens antenna, (**a**) front view; (**b**) side view; (**c**) back view.

**Figure 5:**
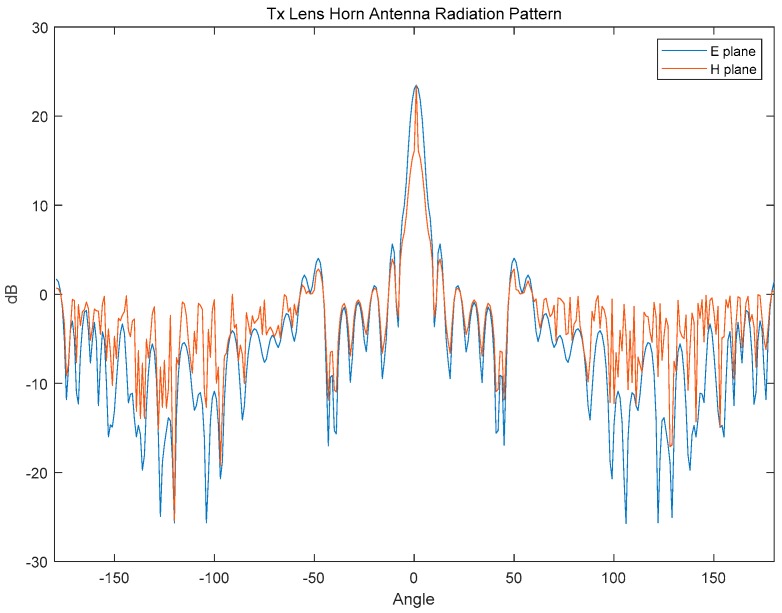
Simulated Tx Lens Horn Antenna Radiation Pattern.

The Tx antenna in the implemented FMCW radar system employs a lens antenna as shown in Figure 4, and Figure 5 shows the simulated Tx antenna radiation pattern. The designed lens antenna has high gain of 25-dBi, side-lobe levels lower than 5-dB, dimensions of 20 cm × 20 cm × 5 cm (including the radome, as shown in Figure 4), and a 4° half power beam width (HPBW). The Tx lens antenna is available for any frequency in the 22 to 25-GHz range, and the implemented FMCW radar system operates at the 24.025 to 24.225-GHz range with a 200-MHz bandwidth.

**Figure 6:**
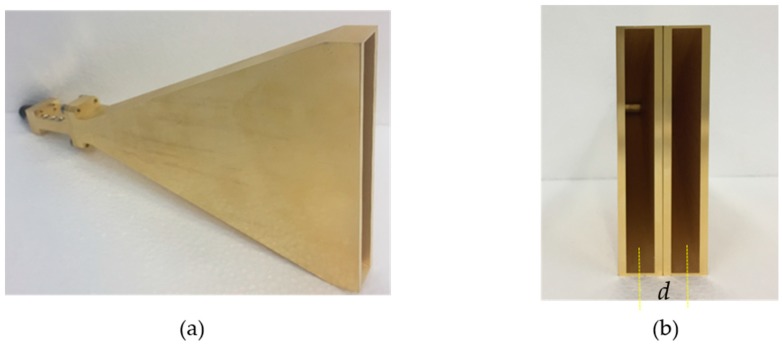
Illustration of the Rx antennas, (**a**) the implemented E-plane sectoral horn antenna; (**b**) two-element Rx antenna array with the distance *d* between the centers of the individual antennas.

Two designed E-plane sectoral horn antennas are utilized as the Rx antenna array in our implemented FMCW radar system, as shown in Figure 6, and the two receiving antennas are located by 1 wave length distance horizontally, resulting in a field of view of ±30° in azimuth.

**Figure 7:**
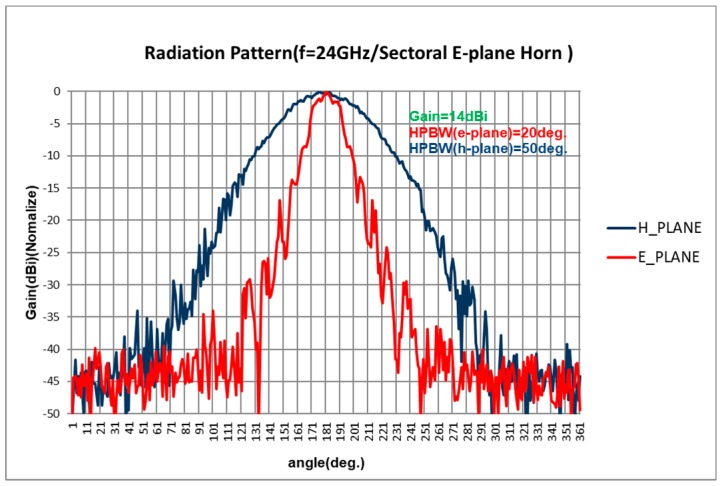
Measured Rx E-plane sectoral horn antenna radiation pattern (normalized).

The antenna spacing *d* of the receiving antenna array determines the field of view (FOV) of the radar systems, and the FOV will be 90° when *d* = *λ*/2*,* and it will be 60° when *d* = *λ*. Generally, for a standard horn antenna, the gain can be improved by increasing the aperture dimensions, and the FOV of the radar systems will be decreased. However, for long-range drone detection, high gain of the received antenna is required. In our implemented system, the designed E-plane sectoral horn antenna is adopted as the receiving antenna. The aperture dimension of the designed E-plane sectoral horn antenna in E-plane is enlarged to provide a higher gain, while the FOV of the radar system is unchanged. The E-plane sectoral horn antenna is designed to have a 14-dBi gain, an E-plane pattern (20°) and an H-plane pattern (50°), as shown in Figure 7.

### 5.2. 24-GHz Transceiver and IF 

**Figure 8:**
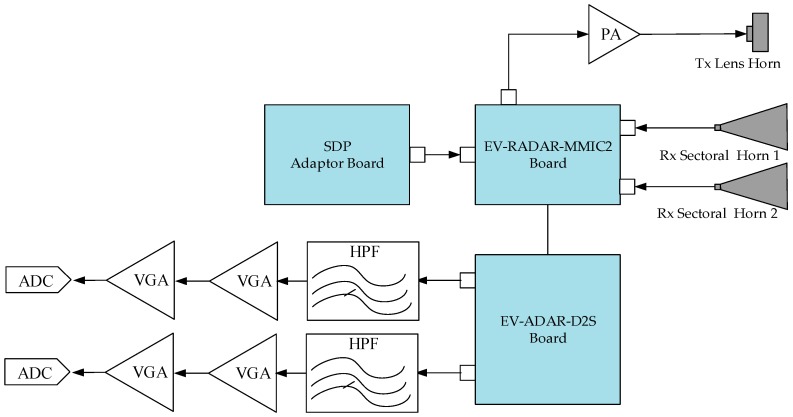
Block diagram of the implemented 24-GHz transceiver and Intermediate Frequency (IF) module.

**Figure 9:**
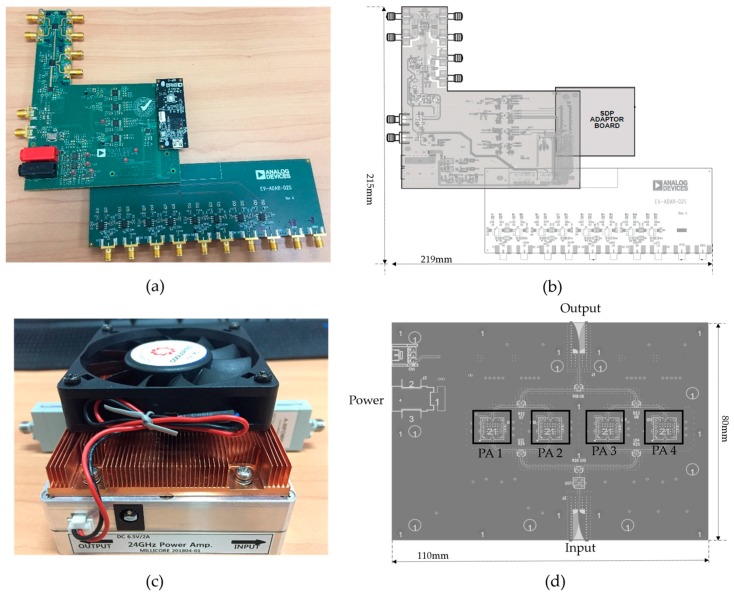
(**a**) Photograph of Adopted Analog Devices evaluation board; (**b**) layout of the Analog Devices evaluation board; (**c**) photograph of the designed 24-GHz power amplifier (PA); (**d**) layout of the designed 24-GHz power amplifier (PA).

The 24-GHz FMCW RF system was implemented with the evaluation board EV-RADAR-MMIC2 from Analog Devices (Norwood, MA, USA), which enables the user to evaluate the performance of a radar chipset comprising a two-channel transmitter chip (ADF5901 designed by Analog Devices), a four-channel receiver chip (ADF5904 designed by Analog Devices), and a fractional-N synthesizer chip (ADF4159 designed by Analog Devices) for FMCW operation. Moreover, the evaluation board EV-RADAR-MMIC2 works with the baseband adapter board EV-ADAR-D2S, which physically attaches to the EV-RADAR-MMIC2. This converts the ADF5904 differential baseband signals to single-ended signals. Signals can propagate through the circuits in differential signaling mode or single-ended signaling mode [28,29]. Differential signaling is a method of signal transfer that uses two signal paths. Whereas the alternative, single-ended signaling, transmits signals between one signal path and a reference ground path, differential signaling transmits signals between two signal paths and a reference ground path. The evaluation board is controlled from a laptop over USB via a microcontroller interface board (SDP adaptor board), which also physically attaches to the main board. Evaluation software provided by the manufacturer provides a user interface from which one can control all the functions of all three chips such as transmit or receive channel enable, FMCW chirp duration, bandwidth and so forth. A block diagram of the implemented FMCW radar system is shown in Figure 8, and the applied boards, EV-RADAR-MMIC2, ADAR-D2S (designed by Analog Devices), and SDP adaptor board (designed by Analog Devices), are shown in Figure 9a,b.

Since the implemented FMCW radar system was developed for long-range drone detection up to 1 km, the Tx signal must be transmitted with a high gain. As shown in Figure 8, the transmission signal generated by the evaluation board, EV-RADAR-MMIC2, is first fed to a designed 24-GHz power amplifier (PA) with 32-dB gain. A photograph of the designed 24-GHz PA is shown in Figure 9c; the PA is mounted with a cooling fan, and the output/input interface employs an end-launch waveguide to a coaxial adapter with a working frequency range of 18-GHz to 26.5-GHz. Figure 9d shows the structure of the designed 24-GHz PA, and the designed circuit consists of four identical single PA chips PA1−PA4. The single PA chip MAAP-011146-STD, developed by MACOM, provides 24-dB gain, 34-dBm output P1dB (P1dB means 1 dB compression point of power amplifiers). In our designed circuit as shown in Figure 9d, four single MAAP-011146-STD chips are in parallel connection to achieve the 32-dB Gain and 38-dBm output P1dB.

**Figure 10:**
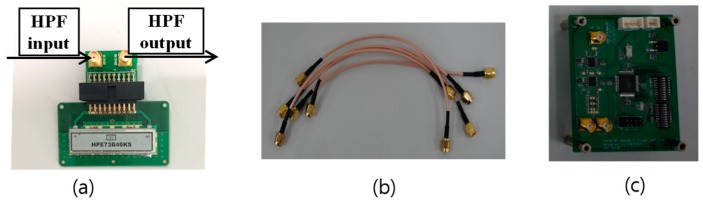
Submodules of IF, (**a**) high-pass filter; (**b**) coaxial cables; (**c**) voltage gain control amplifier (VGA).

For each receiving channel, the output of the adapter board EV-ADAR-D2S is first amplified by the voltage gain control amplifier (VGA), as shown in Figure 10c, with the gain of 21-dB. The gain of the designed VGA covers from −11-dB to 32-dB. 

**Figure 11:**
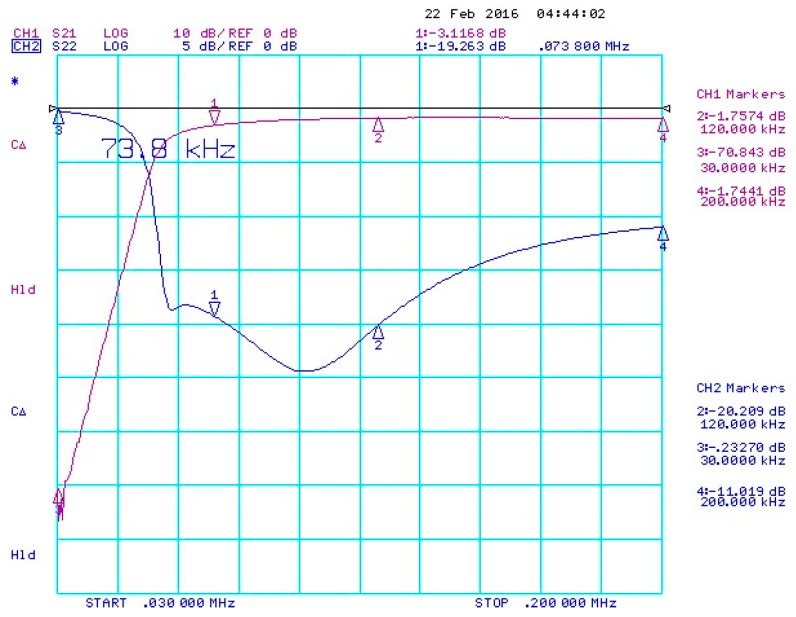
Performance of the high-pass filter.

Since the isolation between the Tx antenna and the Rx antenna array are significantly large with the maximum value of −30-dB, the amplified beat signals from the first VGA are fed to a high-pass filter (HPF), as shown in Figure 10a, with the 3-dB cutoff frequency of 73.8-KHz. Figure 11 shows the performance of the HPF by the values of insertion loss and return loss. The curve for CH1 (S21) presents the insertion loss of the filter along with the frequency, and the frequencies under 73.8-KHz is filtered by the HPF. The curve for CH2 (S22) presents the return loss of the filter along with the frequency, and return loss above 73.8-KHz is very low with about −20 dB. Thus, the designed HPF will filter the frequencies above 73.8-KHz with a very low return loss, and the strong signals affected by the isolations between the Tx antenna and the Rx antenna array will be weakened by the HPF. Following the HPF, the second VGA is applied and then the amplified beat signals are transferred to the data-logging platform. Figure 10b shows the coaxial cables for the connections between the modules.

### 5.3. Data-Logging Platfrom

**Figure 12:**
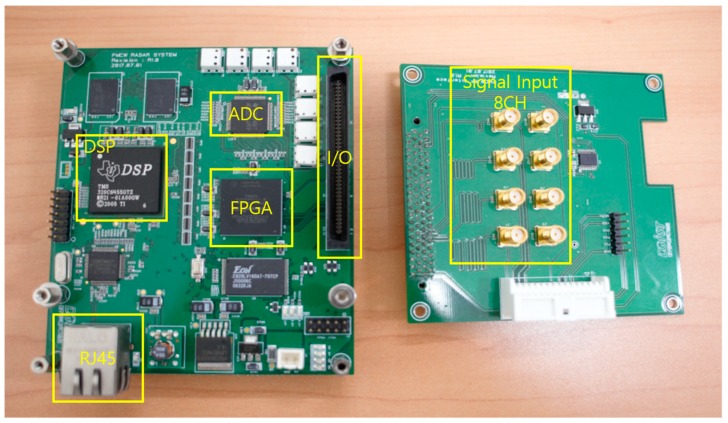
Photograph of the developed data-logging platform.

The data-logging platform was implemented to transfer maximum 8 CH ADC input signals to the PC in real-time, and the 12-bit ADC samples the received beat signals with the sampling frequency of 12.5 MHz. This platform mainly consists of DSP and FPGA chips, namely TI TMS320C6455 and Stratix EP3C25F32. The beat signals sampled by the ADC are first saved in the FIFO of the FPGA and then transferred to the DDR2 SDRAM through a direct memory access mechanism. The saved data can be handled by the DSP chip or transferred to the PC through 1 G LAN communication through the RJ45 interface, as shown in Figure 12, and then the raw radar data saved in the PC is processed by the proposed super resolution algorithm.

## 6. Experiments

This section presents several experiments that were conducted to investigate the performance of the proposed 3D subspace-based algorithm and the developed 24-GHz drone detection FMCW radar system. The received data of the radar system are sampled by the FPGA and DSP board and then processed by the proposed algorithm in MATLAB 2016b with accelerated computing by the graphics processing unit (GPU) of the GeForce 1060 (3 G memory version) graphic card.

**Figure 13:**
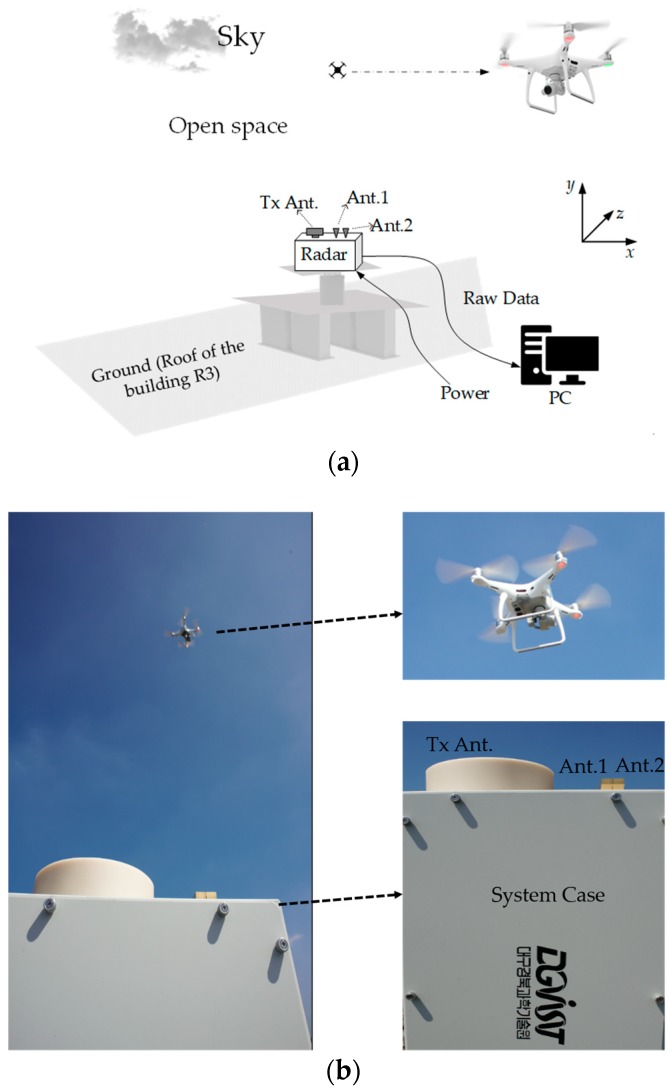
(**a**) Illustration of the experimental set up; (**b**) photograph of the experiments.

The experiments were carried out on the roof of the building R3 of Daegu Gyeongbuk Institute of Science and Technology (DGIST) in breezy and sunny weather, and the experiment scene is shown in Figure 13. The Tx antenna and the Rx antennas were positioned on a designed firm system case, and the 24-GHz transceiver and IF are placed inside the firm system case. The whole radar system was arranged on a steady fixture with a 1.5 m height, and the fronts of all the Tx/Rx antennas were aligned to the horizontal level to ensure the boresight of the antennas was upright. Thus, the modeled Cartesian coordinate system in Figure 1 was established, with the y-axis along the boresight of the Rx antenna array (facing the sky) and the x-axis along the Rx antenna array.

**Figure 14:**
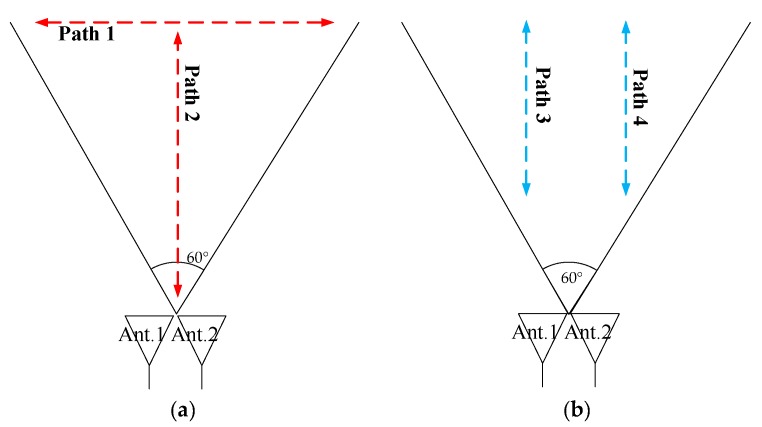
Illustration of the designed flight paths for the drones, (**a**) horizontal flight in Path 1 for Experiment 1, and vertical flight in Path 2 for Experiment 2; (**b**) two vertical paths for Experiment 3.

Four flying paths of drones are depicted in Figure 14, and they are all in the x-y plane. Path 1 was a horizontal line in the height around of 1000 m, and the azimuth angle is limited inside the FOV (±30°) of radar system. Path 2 was a vertical line along the z-axis, Path 3 is along the line *x* = 50 (a path parallel to z-axis), and Path 4 is along the line *x* = −50. The range in the direction of y-axis for Path 2, 3 and 4 is limited from 200 m to 1000 m.

**Figure 15:**
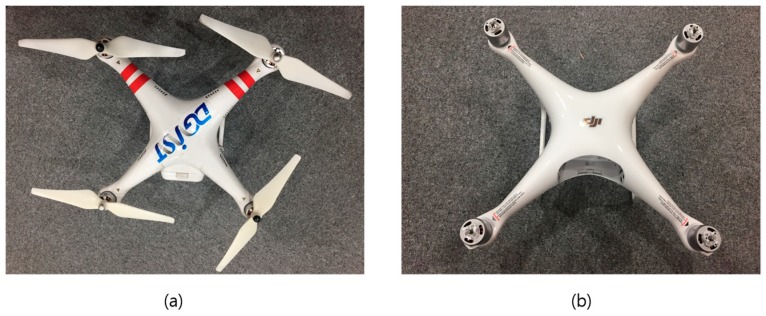
Photographs of drones used in the experiments, (**a**) quadcopter drone 1 (QD1); (**b**) quadcopter drone 2 (QD2).

We chose two quadcopter drones (QD) manufactured by DJI corporation, and photographs of QD1 and QD2 are shown in Figure 15. Three sets of experiments were designed to investigate the proposed algorithm and the implemented FMCW radar system: (1) Experiment 1: QD1 flew along Path 1; (2) Experiment 2: QD2 flew along Path 2; (3) Experiment 3: QD1 and QD2 flew at the same time along Path 3 and Path 4, respectively.

### 6.1. Stationary Clutter Mitigation

In the realistic environment, there is much undesired stationary clutter, such as stationary targets around the radar system, multiple reflections from the walls, and strong Tx/Rx coupling signals, which render drone detection difficult. One subspace projection approach has been proposed for wall clutter mitigation in a through-wall radar imaging (TWRI) system [30]. TWRI system deals with imaging and detection of targets behind walls and in enclosed structures. Generally, wall reflections are often stronger than target reflections, and they tend to persist over a long duration of time. The work [30] proposed a subspace-based wall clutter mitigation approach to mitigate the spatial zero-frequency and low-frequency components which correspond to wall reflections. In our implemented radar system, the reflections induced by stationary clutter are similar with the wall reflections in the through the wall radar imaging radar system. Therefore, we adopted the wall cutter migration technique in [30] to mitigate the stationary clutter induced reflections. For instance, in the detection results without the stationary clutter mitigation approach, the real drone target is masked by the strong undesired signal, as shown in Figure 15a. Since there are many stationary clutter signals, the separation of the signal subspace and the noise subspace in Equation (18) fails, and the drone induced signal is assigned to the noise subspace by the MDL method. Therefore, the preprocessing for stationary clutter mitigation is applied before organizing the EVD for the 3D auto-correlation matrix ***R*** in Equation (18). Following the subspace projection approach in [21], one stationary clutter subspace projection operator ***P****_c_* is utilized to mitigate the stationary clutter induced signals:

(23) $$\hat{R}=P_{c}\tilde{R}$$

where $\tilde{R}=R-me^{T}$, ***m*** is the mean of the columns of ***R***, and ***e***^T^ = [1, …, 1]. More details about the subspace projection approach can be found in section IV of [21]. Since the stationary clutter induced signals are mitigated, the drone induce signals can be separated into signal subspace. The obtained matrix $\hat{R}$ is processed by the EVD:

(24) $$\begin{array}{l} \hat{R}=\hat{U}\hat{\Sigma }{\hat{U}}^{H},\\ \mathrm{where}\;\hat{U}=\left[\begin{array}{cc} {\hat{U}}_{s} & {\hat{U}}_{n} \end{array}\right],\hat{\Sigma }=\left[\begin{array}{cc} {\hat{\Sigma }}_{s} & {\hat{\Sigma }}_{n} \end{array}\right] \end{array}$$

**Figure 16:**
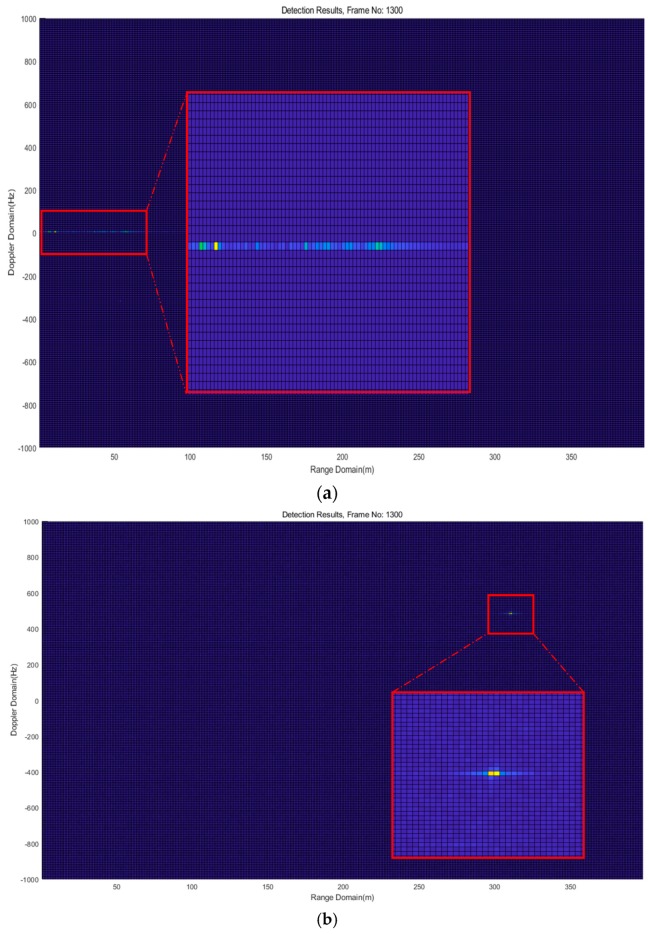
Illustration of the detection results, (**a**) without the clutter mitigation approach; and (**b**) with the clutter mitigation approach.

**Figure 17:**
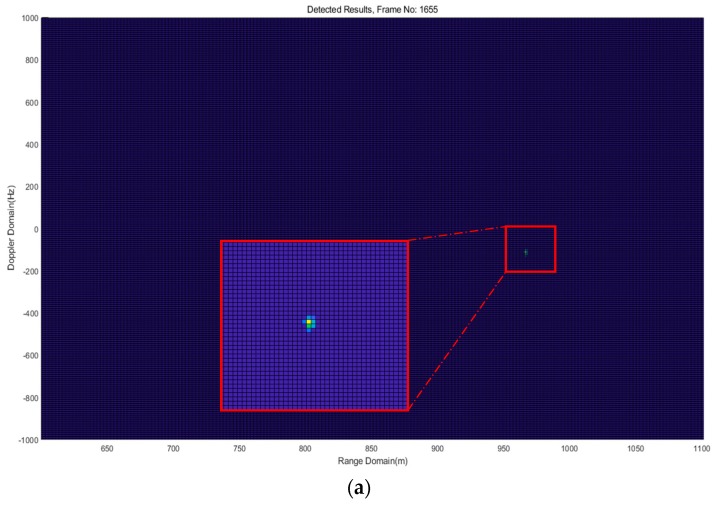

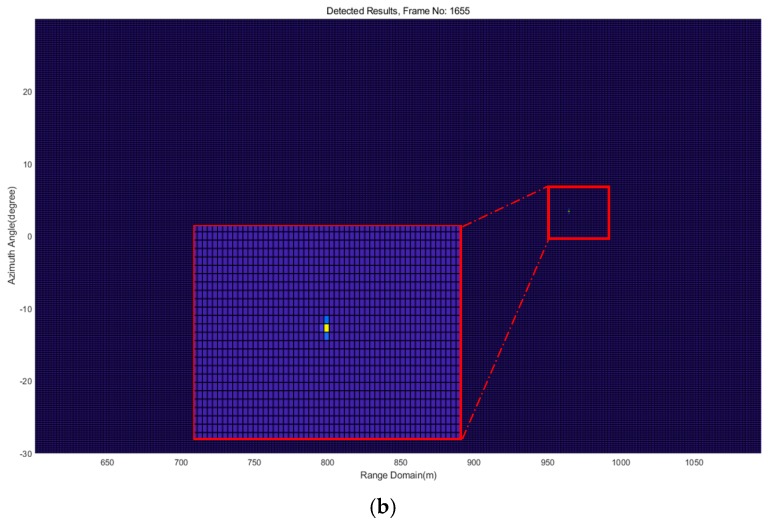
Illustration of one frame of detection results of Experiment 1, (**a**) range-Doppler map; (**b**) range-angle map.

**Figure 18:**
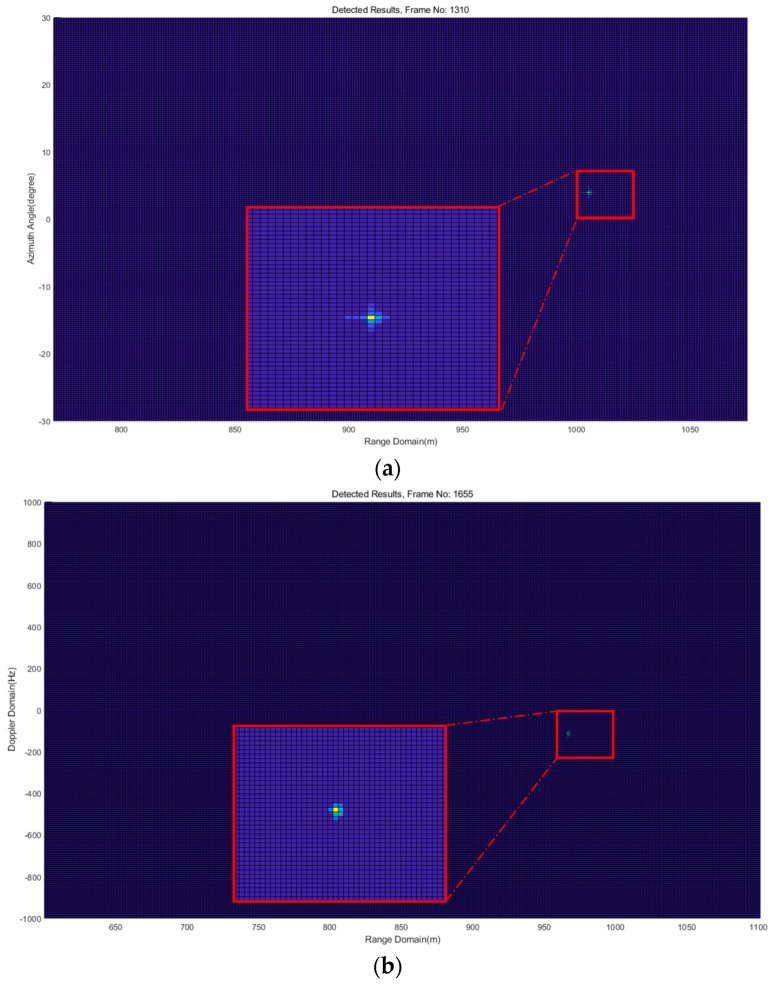
Illustration of one frame of detection results of Experiment 2, (**a**) range-Doppler map; (**b**) range-angle map.

**Figure 19:**
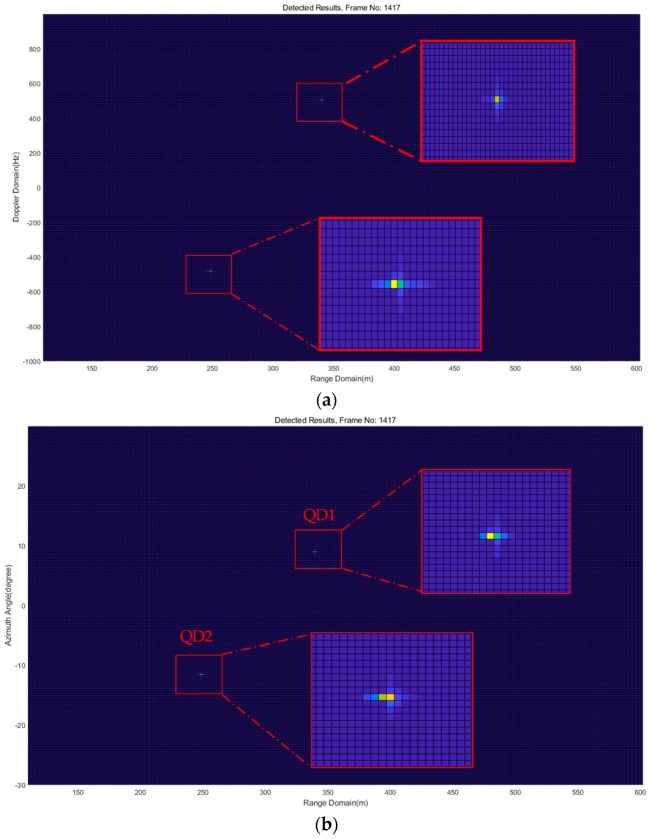
Illustration of one frame of detection results of Experiment 3, (**a**) range-Doppler map; (**b**) range-angle map.

The obtained ${\hat{U}}_{s}$ is utilized for spectrum calculation instead of ***U****_s_* in Equation (20). By mitigating the stationary clutter induced signals with the subspace projection approach, a drone can be successfully detected, as shown in Figure 16 and the results in Figure 17, Figure 18 and Figure 19 of the next subsection.

### 6.2. Experiments Results

Generally, it is hard to represent the 3D pseudo-spectrum in figures. Thus, the obtained 3D pseudo-spectrum is represented as two spectrums: range-azimuth spectrum and range-Doppler spectrum. As explained in the Section 4, there are three indexes {*q_k_*,*w_k_*,*g_k_*} of the *k*-th peak of 3D pseudo-spectrum, and the estimations for ranges, azimuth angles, and velocities of *K* targets will be calculated through the relationships in Equation (19). Once the peaks are detected, the estimations for range, azimuth, and velocity can be calculated.

Figure 16 shows one frame of the detection results of Experiment 1 for QD1 in Path 1, range-Doppler map in Figure 17a and range-azimuth map in Figure 17b. It can be seen that QD1 was flying in the range around 966 m at an azimuth angle of 13.5° and a radial speed of about −0.7 m/s. The calculated absolute speed was around 3 m/s, which approximately equals the value displayed in the controller. One frame of the detection results of Experiment 2 for QD2 in Path 2 is shown in Figure 18. The estimated range was around 1005 m at an azimuth angle of 4.5° and a radial speed of about 3.4 m/s. In Experiment 2, according many detection results, drone detection becomes difficult for the implemented FMCW radar system when the distance of the drone from the radar system is greater than about 1005 to 1010 m. For Experiment 3, the two drones were flying at the same time. One frame of the detection results in Figure 18 shows that QD1 was moving away from the radar system at the range of around 339 m at an azimuth angle of 8.8° and a radial speed of about 3.1 m/s; QD2 was moving award toward the radar system at the range of around 247 m at an azimuth angle of −11.5° and a radial speed of about 3 m/s.

**Figure 20:**
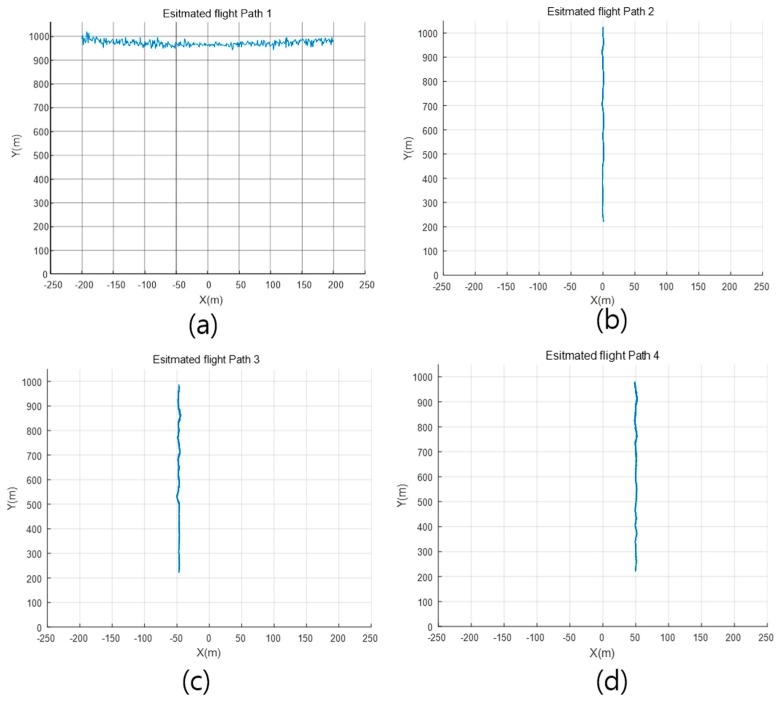
Estimated flight paths, (**a**) estimated path 1; (**b**) estimated path 2; (**c**) estimated path 3; (**d**) estimated path 4.

By analyzing the raw data of all frames for one experiment set, the flight path can be estimated, and the estimated flight paths of all experiment sets are presented in Figure 20. When the drones flew in a location with a large azimuth angle, the detection results were influenced by the narrow beam width of the Tx antenna, as shown in Figure 20a. Since the flight of drones is affected by the wind, it can be seen that drones cannot fly stably above 400 m altitude, but the estimated results can be accepted due to the difficulty of long-range drone detection.

## 7. Conclusions 

A prototype of a 24-GHz frequency-modulated continuous-wave (FMCW) radar system with two sectoral horn antennas and one transmitting lens antenna for the long-range drone detection was presented, and a 3D subspace-based algorithm was proposed for the joint range-azimuth-Doppler estimation of long-range drone detection. In a realistic outdoor environment, three sets of experiments were conducted to detect two quadcopter drones, and the subspace projection approach was utilized to mitigate the stationary clutter. The experiment results proved that the feasible distance of drone detection is up to 1km with the implemented 24-GHz FMCW radar system. Additionally, the effectiveness and performance of the proposed 3D subspace-based algorithm was verified. The proposed algorithm constructs the 3D auto-correlation matrix for lower computing complexity, and avoids the estimated parameter matching for range, azimuth and Doppler. Since the proposed method is still composed of a variety of matrix operations and 3D spectrum searching, it was difficult to implement the algorithm through the hardware. Instead, the processing of the raw radar data with the proposed algorithm is accomplished by MATLAB 2016b in a high-performance PC.

The proposed algorithm is realized in MATLAB and applied with the raw radar data of the experiments, and the future work is to develop the implementation of the proposed algorithm on FPGA and DSP for real time processing. Moreover, the estimation of radar targets is organized the assumption that the radar system remains stationary. It is necessary to develop new signal model and algorithm for the situation when the radar system is placed on a moving platform.

## Figures and Tables

**Table 1:** Comparison of drone classification/detection with the previous works.

	Function of System	Radar System	Parameters (Dimensions)	Detection Range
[14]	Drone calissfication	94 GHz FMCW	Range (1D)	120 m
[15]	Drone calissfication	9.7 GHz CW	Range (1D)	3~150 m
[16]	Drone calissfication	2.4 GHz Pulsed Radar	Range (1D)	~60 m
[17]	Drone calissfication	BirdRad radar	Range (1D)	300~400 m
[18]	Drone detection	24 GHz FMCW	Range/Doppler (2D)	500 m
This work	Drone detection	24 GHz FMCW	Range/Azimuth/Doppler (3D)	Up to 1 km
